# Non-surgical and non-chemical attempts to treat echinococcosis: do they work?[Fn FN1]

**DOI:** 10.1051/parasite/2014071

**Published:** 2014-12-23

**Authors:** Francesca Tamarozzi, Lucine Vuitton, Enrico Brunetti, Dominique Angèle Vuitton, Stéphane Koch

**Affiliations:** 1 Department of Clinical, Surgical, Diagnostic and Paediatric Science, University of Pavia Via Brambilla 74 27100 Pavia Italy; 2 WHO-Collaborating Centre for Clinical Management of Cystic Echinococcosis Via Taramelli 5 27100 Pavia Italy; 3 Gastroenterology and Endoscopy Unit, Besançon University Hospital, Bd Fleming 25000 Besançon France; 4 WHO-Collaborating Centre for Prevention and Treatment of Human Echinococcosis, University of Franche-Comté and University Hospital 25000 Besançon France; 5 Division of Tropical Infectious Diseases, San Matteo Hospital Foundation Via Taramelli 5 27100 Pavia Italy

**Keywords:** Cystic echinococcosis, Alveolar echinococcosis, Non-surgical interventions, Non-chemical treatment

## Abstract

Cystic echinococcosis (CE) and alveolar echinococcosis (AE) are chronic, complex and neglected diseases. Their treatment depends on a number of factors related to the lesion, setting and patient. We performed a literature review of curative or palliative non-surgical, non-chemical interventions in CE and AE. In CE, some of these techniques, like radiofrequency thermal ablation (RFA), were shelved after initial attempts, while others, such as High-Intensity Focused Ultrasound, appear promising but are still in a pre-clinical phase. In AE, RFA has never been tested, however, radiotherapy or heavy-ion therapies have been attempted in experimental models. Still, application to humans is questionable. In CE, although prospective clinical studies are still lacking, therapeutic, non-surgical drainage techniques, such as PAIR (puncture, aspiration, injection, re-aspiration) and its derivatives, are now considered a useful option in selected cases. Finally, palliative, non-surgical drainage techniques such as US- or CT-guided percutaneous biliary drainage, centro-parasitic abscesses drainage, or vascular stenting were performed successfully. Recently, endoscopic retrograde cholangiopancreatography (ERCP)-associated techniques have become increasingly used to manage biliary fistulas in CE and biliary obstructions in AE. Development of pre-clinical animal models would allow testing for AE techniques developed for other indications, e.g. cancer. Prospective trials are required to determine the best use of PAIR, and associated procedures, and the indications and techniques of palliative drainage.

## Abstract (fr)

L’échinococcose kystique (EK) et l’échinococcose alvéolaire (EA) sont des maladies chroniques, complexes, et négligées. Leur traitement dépend de facteurs associés à la nature des lésions, du patient et du lieu de prise en charge. Nous avons réalisé une revue de la littérature concernant les modalités curatives ou palliatives d’interventions non-chirurgicales et non-chimiothérapiques dans l’EK et l’EA. Dans l’EK, certaines de ces techniques, comme l’ablation par radiofréquence (ARF), ont été mises à l’écart après des premières tentatives ; d’autres comme les ultrasons focalisés de haute intensité semblent prometteuses, mais encore au stade préclinique. Dans l’EA, l’ARF n’a jamais été essayée ; toutefois, la radiothérapie et l’hadronthérapie ont été testées dans les modèles expérimentaux, mais on peut douter de leur application en clinique humaine. Dans l’EK, bien qu’on manque encore d’études cliniques prospectives, les techniques de drainage non-chirurgical à visée thérapeutique, comme la perforation, aspiration, injection, ré-aspiration (PAIR) et ses variantes, sont maintenant considérées comme des options valables dans des cas sélectionnés. Enfin, des interventions palliatives percutanées de drainage biliaire guidées par écho- ou scannographie, de drainage d’une cavité centrale surinfectée, ou de pose de stent vasculaire, ont été couronnées de succès. Les techniques de drainage associées à la cholangio-pancréatographie per-endoscopique sont maintenant plus souvent utilisées pour le traitement des fistules biliaires dans l’EK et des obstructions biliaires dans l’EA. Le développement de modèles animaux précliniques permettrait de tester différentes techniques développées pour d’autres indications, comme le cancer, en particulier pour l’AE. Des études prospectives sont nécessaires pour déterminer la meilleure utilisation de la PAIR et des procédures associées, et les meilleures indications et techniques de drainage palliatifs.

## 1. Introduction

Cystic echinococcosis (CE) and alveolar echinococcosis (AE) are chronic, complex and neglected diseases that mostly affect the liver [15]. Their treatment depends on a number of factors, such as location, size and stage of the cysts/lesions, and availability of therapeutic options in each health centre [17]. Despite the wealth of scientific literature on treatment for echinococcosis, the current management of these diseases is based only on poor to moderate quality of evidence and recommendation strength [17]. In addition, therapeutic strategies have been developed over time without systematic and adequate evaluation of their efficacy, effectiveness and safety. This situation derives from the lack of large, longitudinal, controlled studies, which in turn is partly due to the chronicity of the diseases, which require follow-up of many years. The lack of adequate funding, due to echinococcosis’ status as a neglected disease, also makes these costly trials inaccessible [15, 18]. Although the recommended multidisciplinary and stage-specific approach [17] may be available in referral centres, this is often not the case in many endemic countries, where the most affected populations have limited access to diagnosis and therapy, and where the risks associated with invasive procedures may be particularly high. In light of these difficulties, the ideal treatment for active CE and AE should be a procedure that is (i) “technically mature”, (ii) effective on all active CE cyst/AE lesion stages, (iii) safely applicable in resource-poor settings (both technically and economically) and (iv) effective and safe to execute, as confirmed by large, randomized, controlled trials and subsequent long-term follow-up trials. Unfortunately, 40 years since the introduction of benzimidazoles [7, 70], and 30 years since the development of PAIR (Puncture, Aspiration, Injection of a scolicidal agent, Re-aspiration) for the treatment of CE [57], this goal is still remote.

For a long time, surgery has been the only available treatment for CE and AE. In the last decades, however, other options have become available. These options include medical therapy with benzimidazoles, alone or as an adjuvant to invasive treatments, and percutaneous procedures, which may be curative for CE and only palliative, albeit most useful, for AE [17]. A recent international expert consensus has indicated the need for a stage-specific, rational approach to the clinical management of CE patients, and proper multidisciplinary care management, guided by the PNM (parasite lesion, neighbouring organ invasion, metastases) stage of the disease, for AE patients [17, 45]. For CE, uncomplicated active cysts of the liver should be treated, in most cases, by non-surgical options, while surgery should be used when complications are present or in other selected circumstances [17]. Cysts that are asymptomatic and inactive should only be monitored regularly by ultrasound, using the so-called “watch-and-wait” approach [17]. This approach is supported by the observation that CE cysts have a complex course developing over months or years, with recent studies revealing a stage-specific metabolic profile and response to non-surgical treatments [33, 40, 42, 81]. In particular, while univesicular CE cysts (CE1 and CE3a) can often be successfully treated by medical and conventional percutaneous procedures such as PAIR cysts with daughter vesicles (CE2 and CE3b) are most often refractory to these interventions. They could be classified in the group of “complicated cysts” which, like cysts with clinical complications, should most often be treated by surgical operations of various types and complexity, by dedicated percutaneous catheterization techniques, or, although less effectively, by medical treatment alone. For AE, after decades of surgical indication in nearly all patients, the current consensus is to avoid surgery whenever the lesions cannot be removed radically by liver resection [17]. This has opened new opportunities for non-surgical interventions, either percutaneous or perendoscopic, especially for the treatment of bacterial superinfection and/or biliary complications. For both diseases, the incomplete efficacy of the currently available medical therapy with albendazole or mebendazole makes the search for new therapeutic approaches crucial. This is particularly true for AE, for which none of these drugs is parasitocidal but only parasitostatic, and treatment is therefore life-long and burdened by side effects [17, 22, 65, 88]. This is also true for CE, because of the resistance of more than one third of all cysts to medical treatment [81]. Despite preliminary results of the *in vitro* and *in vivo* experimental application of a few anti-infectious or anti-tumour compounds for the treatment of echinococcosis, no drugs have become clinical candidates [38, 88]. Alternative, non-surgical, non-pharmaceutical procedures, such as those used in the treatment of cancer (e.g. radiofrequency and therapeutic ultrasound), could thus become an option, provided that an appropriate pre-clinical evaluation has showed evidence of their efficacy and safety.

Here we present a descriptive literature review of non-surgical and non-medical interventions for CE and AE and their complications, with focus on their state of advancement towards the goal of an “ideal” treatment.

## 2. Materials and methods

We performed a PubMed (MEDLINE) search of the literature on CE and AE, with particular regard but not restricted to, hepatic location, on non-surgical drainage techniques, non-conventional treatments of cysts and non-surgical treatments of biliary complications. The search used the following keywords: *Echinococcus granulosus*, cystic echinococcosis, hydatidosis, hydatid disease, cystic hydatidosis, cystic hydatid disease, *Echinococcus multilocularis*, alveolar echinococcosis, PAIR, catheterization, radiotherapy, radiofrequency, focused ultrasound, endoscopy, cryotherapy, thermotherapy, biliary complication.

We also took into account abstracts selected for communication at the international symposium: “Innovation for the Management of Echinococcosis-2014” [89]. We reviewed the obtained references from 1978; this year was chosen because we were unable to obtain articles on the topics explored before that year, and because it marked the beginning of benzimidazole treatment of CE and AE [7]. When the original paper was either not available or in a language other than English, French, Spanish, Italian, or German, the abstract was used if available. When systematic reviews were available, these were preferentially used to review data on effectiveness and safety of the intervention. When these were not available, case reports and small case studies were used to obtain data on number of patients treated, cyst/lesion characteristics and follow-up.

## 3. Non-surgical drainage techniques

In CE, non-surgical drainage methods include two broad groups of techniques. First, there are techniques that aim at the inactivation of the germinal layer, such as PAIR. Second, there are techniques that allow the evacuation of the entire endocyst.

In AE, the structure of the metacestode, composed of multiple small cysts aggregated in an otherwise fibrotic pseudo-tumoural mass, renders this type of approach completely inefficient. However, non-surgical drainage has become the treatment of choice when the cavity that forms in advanced cases, due to the necrosis of the metacestode in the centre of the lesion, becomes a source of clinical complications, such as chronic pain and bacterial superinfection [10].

### 3.1. Techniques aiming at inactivation of the germinal layer in CE

In 1985, percutaneous aspiration of echinococcal cysts was reported for the first time by Mueller and colleagues in Boston, USA, in one patient [57]. The technique was then refined in the sheep experimental model and used in the following years in endemic areas such as Tunisia, Italy and Turkey [2, 3, 8, 9, 28, 31]. PAIR [93], the most widely used technique, is now recommended by the WHO-IWGE (Informal Working Group on Echinococcosis) for the treatment of CE1 and CE3a cysts of >5 cm and in other selected cases [17]. In cases with giant cysts (>10 cm), permanent catheterization until drainage is <10 mL/day appears more effective than PAIR [33, 55, 84]. In these cases, the overall response rate is >80%, while multivesiculated CE2 and CE3b cysts have a success rate of <40% [33, 42]. A multicentre, retrospective evaluation of the results of PAIR, launched by the WHO-IWGE [27], as well as a meta-analysis performed in 2003 [79], concluded in favour of the long-term efficacy of PAIR in properly selected cases. However, randomized, placebo-controlled clinical trials on the use of PAIR are lacking. A recent Cochrane review on PAIR with or without albendazole for the treatment of uncomplicated hepatic CE [58] could evaluate only two randomized clinical trials comparing PAIR with either albendazole treatment alone [46] or surgery [47], and no other randomized trial has been published since. Both trials were small (30 and 50 patients in total, respectively), but graded as “adequate”, and demonstrated a significantly better efficacy and lower morbidity than that of the treatments they were compared with, suggesting that “PAIR with or without benzimidazole coverage may be comparable or superior to surgery or medical treatment with benzimidazoles alone for uncomplicated hepatic hydatid cysts”, although “data are not sufficient to draw definitive conclusions” [58]. A systematic review of the vast literature on PAIR is beyond the scope of this work, however, an increasing number of single-centre, retrospective studies also show that PAIR is safe and effective when applied to selected CE cases, although the short follow-up, rarely exceeding 3 years, is a common problem. There is, however, general consensus on PAIR showing significantly lower morbidity and mortality rates, duration of hospital stay and costs compared to surgery [27, 80, 84, 94]. Moreover, it has been applied in pregnant women and children [60, 85].

The main complications of percutaneous techniques are biliary communication, infection of the cyst cavity and anaphylactic reactions. The latter risk has been drastically re-evaluated in a recent review of Neumayr et al. [59], which, after examining 5943 percutaneous procedures, found lethal anaphylaxis occurring in 0.03% of procedures. This frequency is not higher than that observed for drug-related anaphylactic reactions. Although technically applicable as an outpatient procedure [49], PAIR needs to be performed in the presence of resuscitation equipment; albendazole peri-interventional treatment is also mandatory [4, 93]. This does not prevent the procedure from being used in endemic areas without sophisticated hospital settings. In fact, PAIR and catheterization have been used to treat 141 cysts in 85 patients, including 6 pregnant women and 6 children less than 5 years of age, in a mission hospital of the remote Turkana region of Kenya, with excellent reported safety outcomes [26]. As is also indicated for surgery, the scolicidal agent in PAIR should, in theory, be applied only after exclusion of communication with the biliary tree. However, published data on the frequency of chemical sclerosing cholangitis in percutaneous procedures are absent, despite mentions of unpublished cases by some experts during scientific meetings. Only one published report from Haddad and colleagues [36] reported a lack of development of sclerosing cholangitis during a 2-year follow-up of three patients with hepatic CE with frank, unobstructed bilio-cystic fistula treated percutaneously with the use of 1% cetrimide and 20% saline as scolicidal agents.

### 3.2. Techniques aiming at evacuation of the endocyst in CE

Several changes to the classic PAIR technique have been described. These have mostly been applied to the treatment of partially solid or multivesiculated cysts, allowing the aspiration of the cyst content and evacuation of the parasite membranes. They present a useful alternative to surgery for those cyst stages poorly responsive to medical treatment and PAIR [17], but no study has assessed their performance in randomized clinical trials. A summary of these reports is presented in Table 1. Although these techniques have been applied safely and with a good short-term success rate, only a limited number of cases have been treated with each technique, and the overall length of follow-up may have been too short to assess the real relapse rate. Clinical experience with the highest number of cases treated using one of such techniques, namely “puncture, drainage and curettage” (Table 1), is reported in a letter from a Chinese group, with a total of 1614 cysts treated in 1409 patients [90]. Although short-term results appeared quite satisfactory, long-term follow-up of patients (10 years after the publication of these cases) has shown a significant number of recurrences (Wang and colleagues, personal communication), which raises some doubt about the value of the technique and calls for well-designed prospective studies.

**Table 1.** Summary of published CE case reports and case series using percutaneous techniques aiming at evacuation of the endocyst.

Technique*	Cysts treated			Drainage length (days)	Follow-up (months)	Success rate**	References
	*N*	Localization	Stage				
Mechanical suction with wide bore catheter	13	Liver	Gharbi type III	7–40 (mean 11.3)	6–24 (mean 15.2)	100%	[55]
D-PAI	184	Liver	137 univesicular, 47 multivesicular	N/A§ (2–7 days hospital stay)	14–215 (median 54)	95% (5% relapse)	[32]
PEVAC	2	Liver	Gharbi type IV	N/R§	4	100%	[70]
	12	Liver	10 Gharbi type II, 2 Gharbi type III	3–128	4–30	100%	[73]
MoCaT	5	Muscle	Gharbi type III	0–54	36–57	100%	[1]
Coaxial catheter technique	17	5 liver, 5 lungs, 2 spleen, 1 kidney 2 peritoneum, 1 retroperitoneum	6 Gharbi type I, 6 Gharbi type II, 2 Gharbi type III, 3 Gharbi type IV	N/A§ (1–2 days hospital stay)	Mean 19.7	94.2% (5.8% relapse)	[30]
Dilatable multifunction trocar	9	Liver	Gharbi type IV	3–13 (mean 11)	1–48 (mean 15)	100%	[37]
Puncture, drainage and curettage	361	Liver and abdomen	N/R§	N/R§	60	99% (1% relapse)	[91] Chinese
Cutting instrument	32	Liver	20 univesicular, 9 multivesicular, 2 infected, 1 calcified	14–35	9–48 (mean 25.5)	100%	[71]

§ N/A, not applicable; N/R, not reported.

* D-PAI, double percutaneous aspiration and ethanol injection; PEVAC, percutaneous evacuation; MoCaT, modified catheterization technique.

** Defined as complete disappearance, solidification or minimal residual fluid component at the end of follow-up.

### 3.3. Non-surgical drainage in AE

The “central cavity” of advanced AE lesions, sometimes inaccurately called “cyst” by radiologists, is not a cyst with an active germinal layer and hydatid fluid, but a semi-liquid mixture of necrotic tissue which may communicate with the biliary tree. Such lesions are at high risk of bacterial superinfection and can cause life-threatening sepsis [12]. Drainage of this necrotic tissue cannot result in metacestode elimination, since the viable and growing metacestode is located at the periphery of the lesion and is inaccessible to puncture. Palliative surgical resection of the AE necrotic lesions of the liver and/or surgical drainage of the central cavity have been, for a long time, the only options for the treatment of such cases [12, 65]. Since the 1980s, progress in percutaneous US- or CT-guided drainage techniques [86] has progressively made percutaneous drainage replace surgical drainage. Unfortunately, in the published literature, there are no available studies specifically designed to evaluate such an approach in AE. The current recommendations to avoid palliative surgery and to choose percutaneous approaches [17] come essentially from the retrospective evaluation of single-centre series of AE patients [10, 12, 19, 25, 43, 63]. The follow-up of individual cases after percutaneous drainage has also shown that, despite the fibrous and calcified nature of the necrotic cavity edges, significant shrinking did actually occur, which could result in reconsidering the resectability of the lesions on CT or MRI images obtained weeks or months after the drainage [10, 11, 53]. Complete radiological disappearance of the lesions has been observed [48]. But no parasitological evidence of cure was given. Conversely, iterative recurrence of necrotic collection and bacterial and/or fungal infection may also occur after percutaneous drainage [12, 68]. The reasons for such opposite outcomes have never been formally studied. Concomitant antibiotic and/or anti-fungal treatment, adapted to the infectious agents isolated in the necrotic material, is necessary. However, we lack facts and figures to give any recommendations regarding specific management procedures, such as technical precautions and length of catheterization, to safely prevent recurrence.

## 4. Non-conventional treatments

Few attempts have been made to test and/or develop alternative techniques to treat CE or AE.

The use of radiofrequency thermal ablation (RFA) for CE has been abandoned after a few disappointing experiences, and no randomized clinical trials have evaluated this technique. High-intensity focused ultrasound (HIFU) appears promising but is still in the pre-clinical phase.

In AE, because of its similarity to malignant tumours, various techniques and doses of ionizing radiation have been studied *in vitro* on *E. multilocularis* metacestode, or *in vivo*, in either experimental models or in single, difficult cases of human AE. Unfortunately, such attempts have been rather disappointing and alternative techniques have received little attention.

### 4.1. Radiofrequency thermal ablation (RFA)

The use of RFA for the treatment of complex hydatid cysts that were unsuitable for medical or percutaneous treatment, as an alternative to more complex evacuation techniques, was first proposed by Brunetti and Filice [14], and subsequently reported by Bastid and colleagues [6] and by Du et al [23]. The idea behind RFA is that as high temperatures (over 60 °C) denature proteins, exposing the cysts to such temperatures should be a relatively simple way to destroy the germinal layer, without resorting to injection and re-aspiration of a scolicidal agent with the associated risks. This technique, borrowed from oncology and widely used to treat liver cancer, is a minimally invasive, imaging-guided procedure in which a needle that conducts high-frequency electrical energy, is inserted into the cyst, leading to heat-mediated necrosis of the surrounding tissue. In the context of CE, particular advantages of RFA would be the easiness of the procedure and the absence of any aspiration/catheterization/evacuation steps. However, there are significant constraints with the procedure, such as the relatively high costs of the equipment and the unsuitability of the technique for the treatment of superficial or peritoneal lesions, or of cysts localized near hollow and vascular structures. Intra-operative RFA, as an alternative or complementary to the usual resection techniques, has also been proposed [62, 96] but is outside the scope of this review.

Brunetti and Filice [14] reported on the treatment of two patients with a total of three large hepatic CE3b cysts with good post-interventional safety and effectiveness. Three more patients with three CE3b hepatic cysts have been treated by the same group after the publication of their first report, with a median follow-up of 24 months (range 6–96 months), but the mid- and long-term efficacy and safety profiles have been rather disappointing. Four patients experienced a relapse within the first 12 months of the procedure, while one patient opted for surgery shortly after the intervention. Complications included the formation of an abscess in one patient and thrombosis of the hepatic vein in a second patient (diagnosed and treated in another centre) [16].

Bastid et al. [6] treated one patient with a large CE2 hepatic cyst, with a follow-up of 6 months, and Du and colleagues [23] treated 63 hepatic cysts with a follow-up of 12 months, but the cyst stage was not reported. Neither work reported mid-term complications or relapses; however, a follow-up of 6–12 months is too short to assess the real success rate of the procedure. A careful examination of hospital records of the patients treated with RFA by Brunetti and co-workers revealed that in all relapsed cases, the structure of the cyst prevented the full ablation of all daughter vesicles (unpublished), and this may explain the high rate of relapses in their cases. Moreover, the structure of cancer tissue and surrounding liver parenchyma (generally cirrhotic as liver cancer often develops as a complication of advanced cirrhosis) is extremely different from that of CE cysts, which may influence the efficacy of the procedure. Importantly, no preliminary *in vitro* or *ex vivo*/*in vivo* animal studies had been conducted before the application of this technique in human CE. Only a later study performed in 2009 on CE cysts excised from sheep liver and lung confirmed the efficacy of the procedure on isolated cysts [50]; however, to our knowledge an *in vivo* experiment on *E. granulosus*-infected animals has never been performed.

From our literature review, it appears that RFA has never been attempted in AE. However, theoretically, some types of AE lesions could be accessible to radiofrequency, such as non-necrotic central lesions, haemangioma-like or metastasis-like early lesions, or fast-growing pseudo-abscess observed in immune suppressed patients [89]. Well-designed, pre-clinical, experimental models are urgently needed to test this type of approach.

### 4.2. High-intensity focused ultrasound (HIFU)

HIFU is a novel, imaging-guided, non-invasive procedure currently approved in Europe for the treatment of uterine fibroids, and with promising applications in other neoplasms such as prostate, liver and breast cancer. In HIFU, ultrasound waves are focused on a small focal zone, causing coagulative necrosis of a small ellipsoid area of tissue, due to hyperthermia and acoustic cavitation.

Unlike other CE treatment options, research on the efficacy of HIFU has progressed in a more logical way: from *in vitro* [98, 101] and *ex vivo* experiments [20, 52, 91], to *in vivo* animal models [51]. Experience in patients is nevertheless lacking. Table 2 shows a summary of the published work on this topic.

**Table 2.** Summary of published work on the efficacy of experimental HIFU against *E. granulosus*.

Target	Summary of the procedure	Results	References
Cysts *ex vivo*	Cysts treated with 150 W or 250 W sound power respectively.	Detachment and disruption of the germinal layer.	[90] Chinese
PSC *in vitro*	PSC suspension treated with different combinations of sound power (0–250 W) and time (5–60 s).	Dose-dependent damage and death of PSC; growth inhibition and mortality of survived PSC cultured *in vitro*; inhibition of infectivity of survived PSC in secondary infection mouse model.	[100]
Cysts from mouse secondary infection *ex vivo*	Three groups, treated with 4 W, 9 W or 13 W sound power for 1 min.	Damage of the laminated and germinal layers.	[52] Chinese
PSC *in vitro*	PSC suspension treated with 100 W acoustic power for 5–60 s with or without SAP.	Dose-dependent damage and death of treated PSC, enhanced in the presence of SAP.	[97]
Cysts *ex vivo*	Cysts treated with 100 W sound power for 3 s each area for three times at a scanning speed of 3 mm/s with or without injection of SAP or UCA alone or in combination.	Change in ultrasound echo pattern after treatment; increased mortality rate of PSC after treatment HIFU alone < SAP or UCA-aided HIFU < SAP + UCA-aided HIFU; damage of the laminated and germinal layers.	[20]
Cysts transplanted IP in rabbit *in vivo* model	Cysts transplanted IP in rabbits after ± injection of SAP or UCA or both; HIFU applied as in [11].	Change in ultrasound echo pattern after treatment; increased mortality rate of PSC after treatment HIFU alone < SAP or UCA-aided HIFU < SAP + UCA-aided HIFU; damage of the laminated and germinal layers; no pathologic effects on rabbit skin.	[51]

PSC, protoscolices; SAP, superabsorbent polymer; UCA, ultrasound contrast agent.

In the context of CE, HIFU would offer the clear advantage of non-invasiveness, which would reduce or avoid some of the risks associated with percutaneous treatments and surgery, in particular, infection and biliary complications. Preliminary experimental studies seem to show better efficacy when the cyst content density is increased by injection of a superabsorbent polymer and/or ultrasound contrast agent, which reduce the rapid dispersion of heat in the fluid content (Table 2). Although this may raise doubts about the possible applicability of HIFU on all cyst stages, it may nevertheless look promising for the treatment of complex and partially solid cysts, which are also the most difficult to treat with other non-surgical interventions. Other major constraints are the high cost of the equipment, which is not portable, the duration of the procedure and the need for general anaesthesia.

Only one case of AE treated with HIFU has been published, but no details have been given on the case, the mid-term outcome or the long-term outcome [99]. The efficacy of low-power and high-power multifrequency focused ultrasound (MfFU) in the treatment of AE has been studied in experimental mice with *E. multilocularis* subcutaneous infection [95]. Significant damage was observed in AE lesions, more severe in the high-power group (10 W + 11 W + 10 W once for 5 min); protoscolex survival was also lower in the highly irradiated lesions. However, 60% of protoscoleces were still viable after this single irradiation. The efficacy of HIFU in killing *E. multilocularis* deserves further investigation before any pre-clinical studies can be carried out.

### 4.3. Radiotherapy

Historically, the efficacy of radiotherapy on echinococcosis was studied as early as 1904, and results were immediately controversial (Diaz de Quintana, 1904 and Dévé, 1904, cited in [64]). In 1927, after 20 years of controversial experimental and clinical results, Dévé published a Review in the *Presse Médicale* where he stated, “the hydaticidal dose seems to be around or above 20,000R; this dose was administered *in vitro*, without filter, on isolated scoleces; in human radiotherapy, it is absolutely impossible to reach safely such a dose in the depth of organs. Radiotherapy of hydatid cysts still constitutes a mere therapeutic illusion” (in [64]). In 1932, Perrin [64] summarized the studies performed in AE in his MD thesis: “as it stands for now, we consider that radiotherapy is inefficient in the treatment of alveolar echinococcosis”.

Since Perrin’s MD thesis, there have been no systematic studies on the use of radiotherapy in AE. A few clinical attempts in severe cases resistant to all treatments, especially in bone echinococcosis, have been published. However, in several reports from countries where both CE and AE are endemic, distinctions between CE and AE were not clearly made, and long-term follow-up is lacking.

In AE, experimental studies of ionizing radiation were conducted on *in vitro*, cultured *E. multilocularis* vesicles by Pohle and co-workers [66], and on AE cysts *ex vivo* by Zhou et al. [100]. In the first study, if we consider short-term results, the efficacy was not convincing. Although there was a marked decrease in the proliferation and growth of *E. multilocularis* upon long-term analysis, and irradiation led to metabolic impairment of *E. multilocularis* metacestodes, no definite parasitocidal effect was achieved [66]. In the second study, which compared X-ray and carbon-ion irradiation, the effects on the cysts were more impressive, with an LD50 of 28.5 Gy for X-rays and 15.5 Gy for carbon-ion irradiation. Degeneration of the cysts was associated with evidence of mitochondrial DNA damage and evidence of apoptosis, as measured by Caspase 3. Morphological changes were clearly observed in protoscoleces and were more marked after carbon-ion irradiation [100]. In rats infected with *E. multilocularis,* which received 6-MeV radiotherapy at 20 Gy/8 f, 40 Gy/8 f and 60 Gy/8 f, once every 3 days for a total of eight times, inhibitory rates on parasite vesicle growth were 50%, 72% and 82%, respectively. However, despite the fact that injury to the germinal layer was significant and most severe in the high-dose group, there was no complete elimination of the metacestode [100]. In addition, as already stressed by Dévé one century ago, the doses administered both *in vitro* and experimentally *in vivo* are likely not compatible with their use for the treatment of human patients. The clinical case reported by Bao et al. [5] does not indicate, precisely, the irradiation doses and the patient’s follow-up details. In a case published by Ulger et al. [83], irradiation consisted of an external beam radiation dose of 25 Gy (250 cGy/day, 5 days/week) by linear accelerator with 3D conformal radiation. The authors report that after one year, the patient was free of chest pain and his disease was stable; after surgical resection of an associated brain metastasis, the patient “recovered well from brain surgery and was free of symptoms in the sternum area” [83]. However, the actual species of metacestode involved in this case of “cystic echinococcosis caused by *E. multilocularis*”, as stated by the authors, is doubtful, and the authors’ conclusion reported that “a practical and easy method: RT for *E. multilocularis* infection of the sternum as an alternative treatment modality” has been strongly criticized [34]. In this regard, the complete, long-term history of a Swiss patient, whose AE brain metastasis was treated in Austria by gamma-knife radiosurgery, is exemplary [75–77]. After combined treatment, using mebendazole and interferon gamma started at diagnosis in 1992, in 1994 the treatment was switched to albendazole and the gamma-knife procedure was done in two sessions instead of open brain surgery. Gamma-knife surgery was apparently successful, with an impressive shrinkage of the lesions after a temporary major inflammation of the concerned areas. At a follow-up visit 3 years after gamma-knife radiosurgery, the polycystic lesion, the peri-focal oedema and the neurological symptoms had all markedly decreased. In 2012, 20 years after diagnosis and 18 after gamma-knife surgery, the patient, lost to follow-up since 2009, was re-hospitalized and died of respiratory insufficiency [54]. Albendazole had been withdrawn 4 years after the gamma-knife procedure because of chronic hair loss. Histology of the brain, the lung and the liver revealed large cystic lesions with eosinophilic brood capsules and protoscoleces (i.e. definitely viable AE lesions). Indeed, Gripp, Ernst and Pohle rightly stressed that, because of the doubtful efficacy of radiotherapy from the available experimental studies, and in the absence of reliable pre-clinical data, case reports on clinically successful radiotherapy treatments in AE must be viewed with caution [34].

## 5. Non-surgical drainage techniques of biliary complications

### 5.1. Cystic echinococcosis

Biliary communication accounts for up to 60% of hepatic hydatid cyst complications, and is associated with increased morbidity and hospitalization time [69]. Factors predictive of the presence of biliary communication include cyst size >7.5 cm, presence of daughter vesicles or of a thick fibrotic and calcified wall, localization near the porta hepatis, disease recurrence and signs/symptoms of biliary involvement [69].

The choice of treatment of such complications depends on many factors, such as size and location of the cyst, characteristics of the cyst wall and the experience of the health centre. Traditionally, surgery has been the most common approach. However, non-surgical approaches are increasingly used, alone or in conjunction with surgery, although no prospective clinical trials have been conducted (or at least published) on their efficacy and safety. The most common non-surgical technique used in biliary complications is ERCP (endoscopic retrograde cholangiopancreatography) with endoscopic sphincterotomy and nasobiliary drainage, but other techniques have also been reported.

The management of bilio-cystic fistulas has never been evaluated in randomized clinical trials. Two recent systematic reviews addressed the efficacy and safety of ERCP treatment on cysto-biliary communications [24, 69]. These analyses graded as Level IV and Grade C the evidence of ERCP use in the following cases: (i) pre-operatively, allowing the definition of cysto-biliary relations and treatment of biliary obstruction, permitting the performance of elective surgery; (ii) post-operatively, decreasing the incidence of the development of external fistulization and biliary complications by avoiding the need for surgical revision of the bile duct; (iii) post-operatively, allowing definitive cure as the only intervention when complete evacuation of the biliary tract and cyst content is possible; (iv) post-operatively, facilitating the diagnosis and treatment of obstruction, cholangitis and external biliary fistulae. Other non-surgical techniques that successfully treated biliary complications, especially in patients where ERCP failed, have also been published in case reports, and are summarized in Table 3. A stepwise approach was presented by Benazzouz and colleagues at the international symposium “Innovation in the Management of Echinococcosis” [89]. The authors treated 47 hydatid cysts with biliary rupture with an 80.8% success rate without the need for surgery using the following approach: in the case of occult fistula, the treatment was based on percutaneous drainage alone with a large bore catheter, associated with endoscopic biliary clearance in case of failure. In the case of frank fistula, the first-line treatment was endoscopic alone, associated with percutaneous drainage in case of failure.

**Table 3.** Summary of non-surgical, non-ERCP techniques for the treatment of cysto-biliary fistula in hepatic CE.

Technique	Number and type of cyst/lesion	Drainage (days)	Follow-up (months)	References
Percutaneous transhepatic endobiliary drainage	1 Gharbi type IV ruptured and obstructing bile tract	7	30	[41, 73*]
Radiofrequency thermal ablation	1 post-surgical cavity with cutaneous and biliary fistulae	N/A§	9	[81]
N-Butyl 2-Cyanoacrylate embolization	1 post-catheterization cavity with persistent biliary drainage	N/A§	3	[21]
Percutaneous alcohol sclerotherapy after balloon occlusion of fistula	1 cyst (stage not reported) ruptured and obstructing the bile tract, treated with catheterization	24	0	[86]

§ N/A, not applicable.

* Full paper or abstract not available; data refer to reference [41].

Finally, Zeybek and colleagues explored the factors that predict spontaneous closure of cysto-biliary fistulae [97]. In a case-control study of 46 patients with post-operative biliary fistula, they found that a maximum post-operative biliary drainage volume of <102 mL was the only significant predictor of spontaneous closure, although the stratification by type of intervention was not carried out.

### 5.2. Alveolar echinococcosis

Jaundice is the main symptom at the time of AE diagnosis in 25–45% of symptomatic patients, depending on the endemic area and late or early diagnosis. Biliary complications occur in 45% of patients during follow-up [12, 67]. Bacterial cholangitis is a major issue in this disease: in addition to biliary tract obstruction by the invading metacestode, chronic infection of the bile ducts may be responsible for obstruction by biliary stones, which then increases the risk of bacterial infection and leads to secondary biliary cirrhosis [29]. Surgery is the treatment of choice for patients with cholangitis as long as it is curative [17]. However, the curative resection rate is low at the time of diagnosis (20–40%) because of the location and size of the AE lesions, especially in those patients with biliary complications [12]. When surgery is not curative, radiological or endoscopic drainage is an efficient and less invasive alternative to palliative surgery [10, 19, 43]. Biliary drainage can be required before, instead of, or after surgery. As for percutaneous drainage of necrotic cavities in AE lesions, data on percutaneous biliary drainage are very scarce. Some case reports have been published but the main information comes from reviews and expert opinions.

#### 5.2.1. Radiologic drainage: Percutaneous transhepatic biliary drainage (PTBD)

The first case of use of PTBD in AE was reported in 1994 by Bret and colleagues, from Lyon, France [13]. Following this first experience, in the French cohort reported by Bresson-Hadni et al. [10], PTBD was successfully used in 13% of patients to avoid palliative surgery or as a bridge to curative surgical treatment. Radiologic interventional procedures are very helpful in cases of cholangitis related to biliary tree infiltration by parasitic tissue and the associated fibro-inflammatory reaction. In such palliative situations, with hilum involvement leading to cholangitis, a good option is to percutaneously place a drain in a dilated intra-hepatic bile duct, and to push the drain proximally through the parasitic stenosis, then distally through the papilla and finally into the duodenum to obtain external/internal biliary drainage. Such drains may be maintained for years and, coupled with life-long benzimidazole therapy, they may allow prolonged survival in AE cases that were initially very severe [10]. However, the frequent communication between the necrotic part of the parasitic lesion and the biliary tree may cause obstruction of the drain by the migration of necrotic plugs. For this reason, the external-internal biliary drain must be removed and changed regularly every 3 months. PTBD should be external-internal into the duodenum through the papilla in order to avoid bicarbonate loss. Nevertheless, some morbidity has been described, such as skin burn, cholangitis, snatched drain and haemorrhage [10].

#### 5.2.2. Endoscopic drainage: Endoscopic retrograde cholangiography (ERCP)

ERCP should not be used as a diagnostic tool if non-invasive tools to explore the biliary tract are available, but should be the first therapeutic option. ERCP represents a therapeutic alternative to PTBD. The aim of this procedure is to treat jaundice and/or cholangitis while avoiding the discomfort of external drainage. While ERCP for the treatment of liver CE is well described (see Sect. 5.1.), data for AE are scant. Only a few case reports have been published since the first reported case in 1991 [35, 39, 61, 78], but all suggest that a combination of endoscopic and drug treatment is safe and may be effective in managing the obstruction of the biliary tree by *E. multilocularis* metacestode; this represents an interesting alternative to palliative surgery. From the European experience on 30 patients in 12 centres, analysed by Ambregna and co-authors and presented at the international symposium “Innovation in the Management of Echinococcosis” [89], several technical points arise: (i) the necessity of extracting stones with a Dormia basket and intense normal saline lavage before stent placement to avoid septic complications; (ii) the need for several endoscopic hydrostatic balloon dilations to enlarge the bile ducts because strictures are extremely rigid and narrow in AE; and (iii) the need for several plastic stents to caliber the biliary strictures and to reduce the number of procedures. The more biliary stents placed to caliber bile ducts, the longer it is possible to postpone the next procedure without cholangitis recurrence. Nasobiliary drainage has been reported in the past [35, 39], but biliary stents are more comfortable for the patient. Moreover, saline lavage and the use of a Dormia basket allow extraction of fragments of the metacestode together with the sludge and the biliary stones, therefore helping to confirm the parasitic aetiology of the disease [34]. In any case, endoscopic treatments never impair further surgical management, including liver transplantation.

Several technical problems remain unsolved. Removable biliary stents (fully covered self-expandable metallic stents) were designed for distal biliary strictures. They are used to treat biliary stricture or biliary leaks after surgical injury of the biliary tree under the hepatic bile duct convergence or for biliary strictures caused by chronic pancreatitis in the lower biliary tree. These fully covered stents cannot be used in the upper biliary tree, because they may cause obstruction of the lateral bile ducts. The self-expandable, metallic stents which are used for palliative treatment in cholangiocarcinoma of the upper biliary tree, are uncovered and non-removable. However, AE is a “benign” parasitic disease in patients with longer survival time. Therefore the use of several “old type” plastic stents (such as a bundle), which are less easy to place but are completely removable and do not obstruct secondary biliary ducts, may be more suitable. Combination of biliary stent and oral ursodeoxycholic acid or antibiotics is inefficient to avoid stent obstruction in the treatment of pancreatic cancer [44], but may be useful in AE, as advised in the WHO consensus, and other expert opinions, because stone production by chronic cholangitis could theoretically be decreased using this approach [17, 88].

## 6. Discussion

The various treatments for CE have evolved over decades without systematic assessment of their efficacy and effectiveness in randomized trials. This is partly due to the chronicity of the disease, which requires a follow-up of many years to evaluate relapse rates, and to its complexity, which makes it very difficult to design and implement randomized trials stratified by cyst characteristics. The lack of adequate funding due to neglect of CE [15, 18] compounds these difficulties.

As a result, different techniques have been introduced and are “technically mature”, showing at least short-term efficacy, but no conclusion can be drawn on their real performance. In addition to the imperative need to assess the efficacy of different treatment options in randomized trials, future studies should also take into consideration the peculiarities of CE endemic countries, where advanced healthcare facilities are often lacking, or are not available to the majority of the population. A non-invasive procedure with very low relapse rates, as assessed over many years of follow-up, and applicable in all cyst stages, would be ideal. In reality, the long-term effectiveness on the one hand, and short-term risk of complications on the other, should be taken into account. PAIR and catheterization are considered effective and safe treatment options for unilocular hepatic cysts, however, they have limited efficacy in complex or complicated cyst stages. Modified percutaneous techniques such as MoCaT (Modified Catheterization Technique) or PEVAC (Percutaneous Evacuation) (see Table 1) seem more promising in this regard but need long-term evaluation. Less explored techniques, such as RFA, may also be further evaluated for the treatment of complex hepatic cysts. However, pre-clinical studies, such as that conducted by Lamonaca and colleagues [50], should be performed, with special focus on the treatment of complex hepatic cysts. In any case, all percutaneous techniques are invasive, with different levels of complexity, and the need for the development of alternative treatments applicable in remote, resource-poor areas remains urgent. In this regard, HIFU certainly deserves particular attention.

In AE, low expectations should be maintained on radiotherapy, despite the currently available, sophisticated equipment and the improvement of treatment protocols used in cancer. Immuno- or chemo-radioisotope therapies have not been fully investigated, and deserve more attention. As of 2014, percutaneous drainage for the management of necrotic central cavities in advanced lesions and per-endoscopic drainage for obstruction of the biliary tree have nearly completely replaced palliative surgery. Long-term evaluation of their use, as well as technical improvements to better address the specific problems posed by the pseudo-tumour lesions of hepatic AE, are certainly needed.
